# Profiles of behavioral health service utilization among youth involved in the juvenile legal system

**DOI:** 10.1186/s40352-026-00416-1

**Published:** 2026-04-29

**Authors:** Kaitlin Piper, Tasfia Jahangir, Elizabeth Van Alstine, Laura Tyrone, Aanya Ravichander, Kaitlin Sheerin, Crosby Modrowski, Kathleen Kemp

**Affiliations:** 1 Rollins School of Public Health, Emory University, Atlanta, USA; 2 Warren Alpert Medical School, Brown University, Providence, USA; 3 Salve Regina University, Newport, USA

**Keywords:** Juvenile legal system, Behavioral health services, Adolescent health, Barriers to care

## Abstract

**Background:**

Juvenile legal-involved youth (JLIY) experience behavioral health conditions at more than twice the rate of their peers yet face significant barriers to accessing care. These barriers span predisposing (demographic and social), enabling (logistical and resource-related), and need-based (clinical) factors. To better understand how JLIY navigate the fragmented behavioral healthcare system, we examined their use of services across eight different sectors, including formal treatment settings, non-specialized systems (e.g., schools and courts), and informal community supports.

**Methods:**

We surveyed 100 caregiver–youth dyads enrolled in a juvenile court diversion program, all of whom had youth with documented behavioral health needs. We collected information on caregiver and youth demographics, behavioral health symptoms, treatment barriers, motivation for treatment, and service utilization. Latent Class Analysis (LCA) was used to identify distinct patterns of youth behavioral health service utilization across eight care sectors. Multinomial logistic regression was then conducted to examine how predisposing, enabling, and need-based factors, guided by the Andersen Behavioral Model of Health Services Use, predicted class membership.

**Results:**

On average, youth accessed services in 4 of the 8 behavioral health care sectors. Service sectors utilized by JLIY included school-based support (86%), outpatient care (67%), community network supports (61%), crisis services (59%), general healthcare (58%), juvenile legal services (32%), inpatient care (26%), and residential treatment (11%). Latent Class Analysis revealed three distinct service use profiles: (1) low-intensity, school-centric users; (2) high-intensity, multi-sectoral users; and (3) moderate-intensity, community-based users. Class membership was significantly associated with child welfare involvement, court-mandated treatment, caregiver trauma exposure, caregiver motivation for youth treatment, and youth symptom severity.

**Conclusion:**

JLIY navigate a wide range of behavioral health services, often in fragmented or reactive ways. While individual need was a strong predictor of service use, enabling factors such as caregiver influences and system mandates also played a critical role. The reliance on school-based services suggests systemic gaps, and extensive multi-sector involvement may reflect lack of integration across systems. Findings have implications for policy, including the need to strengthen cross-system coordination among juvenile legal, school and behavioral health systems; expanded family-centered service navigation; and improved access to community-based care before needs escalate.

## Introduction

Over half a million youth enter the juvenile legal system (JLS) in the United States each year (Puzzanchera, 2021). Juvenile legal involved youth (JLIY) deal with behavioral health conditions (mental health and substance use disorders) at a rate of 50–70% (Abram et al., 2007; Beaudry et al., 2021; Burke et al., 2015; Teplin et al., 2002; Wasserman et al., 2010), which is over twice the rate of psychiatric conditions in the general adolescent population (McCance-Katz, 2019; Merikangas et al., 2010). Behavioral health concerns among JLIY are also often complex and co-occurring, with approximately 60% experiencing both mental health and substance use conditions (Shufelt & Cocozza, 2006). Common mental health disorders in this population include disruptive or conduct disorders, anxiety disorders, mood disorders, and post-traumatic stress disorder, while common substance use concerns include cannabis, alcohol, and prescription drug misuse (Dennis et al., 2019; Dierkhising et al., 2013; Scott et al., 2019; Shufelt & Cocozza, 2006).

Among JLIY, behavioral health conditions are often exacerbated by system involvement (Jackson et al., 2019; Schweer-Collins et al., 2024) and are frequently accompanied by other socially complex needs, such as housing instability, family trauma, and limited access to care (Kemp et al., 2025; Kinner et al., 2014). The intersecting challenges of behavioral health issues, complex social needs, and involvement in the juvenile legal system are especially acute for youth from racial, ethnic, sexual, and gender minority groups, who face compounded risks due to social marginalization, systemic discrimination, and structural disadvantage (Kemp et al., 2025; Spinney et al., 2016, 2018). Failure to treat behavioral health conditions during childhood and adolescence contributes to higher rates of rearrest and recidivism for JLIY throughout their lifetime (Hoeve et al., 2013, 2014; Schubert et al., 2011; Van der Put et al., 2014), as well as long-term health complications such as early mortality (O’Connor et al., 2023; Ruch et al., 2021), trauma and suicidal ideation (Abram et al., 2007; Nolen et al., 2008; Teplin et al.,; 2021; Wasserman & McReynolds, 2006), and chronic illnesses (Merrick, 2019).

The juvenile legal system (JLS) has a unique opportunity to connect this high-risk group of adolescents with behavioral health treatment (Baker et al., 2021, 2022), which directly aligns with the JLS’s original purpose to prevent adolescents from future involvement in the adult criminal legal system ("Juvenile Justice and Delinquency Prevention Act of 1974," 1974). However, the process for providing behavioral health treatment to JLIY is often complex (Kemp et al., 2025), requiring JLS to screen for behavioral health needs, make care referrals, and coordinate care with community-based providers (Belenko et al., 2017, 2022; Nelson et al., 2024). Even when these processes are in place, the JLS often has limited influence over whether youth and their caregivers ultimately initiate treatment. In some cases, court treatment mandates may increase service entry, but treatment accessed under coercive conditions may not reflect family readiness, youth engagement, or sustained participation in care. These challenges are further compounded by the complexity of cross-system coordination and broader structural barriers to behavioral health care (Chuang & Wells, 2010; Lu et al., 2021; Scott et al., 2019). As a result, screening and referral efforts in the JLS have had limited success: fewer than 10% of justice-involved youth with substance use treatment need receive formal community-based treatment, and fewer than 20% receive formal mental health treatment (Burke et al., 2015; Dennis et al., 2019; Liebenberg & Ungar, 2014; Wasserman et al., 2021). This service gap is particularly concerning given that recidivism among justice-involved youth remains substantial, with many youth experiencing rearrest or renewed system involvement following initial contact with the legal system (Hoeve et al., 2013, 2014; Schubert et al., 2011; Van der Put et al., 2014). As such, improving linkage to behavioral health treatment may be important not only for addressing unmet clinical need, but also for reducing recidivism and supporting the broader rehabilitative goals of the juvenile legal system.

### Factors shaping behavioral health service utilization among JLIY

To better understand the factors shaping behavioral health service access and use among JLIY, the Andersen Behavioral Model of Health Services Use provides a useful conceptual framework for identifying multilevel barriers and facilitators (Andersen, 1995). The model organizes influences on service utilization into three core domains: predisposing, enabling, and need factors. *Predisposing factors*, such as demographic and social characteristics that exist prior to the onset of illness, can shape an individual’s inclination to seek care. JLIY are disproportionately drawn from communities experiencing complex social and structural challenges, and are overrepresented among gender, sexual, racial, and ethnic minority groups (Kemp et al., 2025; Puzzanchera, 2021). Many have had prior involvement not only with the JLS but also with the child welfare system, reflecting intersecting forms of systemic disadvantage (Herz et al., 2021). For these youth, behavioral health often takes a back seat to more immediate concerns such as housing instability, financial hardship, educational disruption, and family insecurity (Almquist & Walker, 2022; Baglivio et al., 2014; Wolff et al., 2018). Furthermore, research has identified persistent disparities among JLIY from minoritized backgrounds in the prevalence of behavioral health disorders, patterns of care-seeking, and the likelihood of detection and treatment by service providers (Rawal et al., 2004; Teplin et al., 2005; Webb et al., 2025).

Importantly, barriers to behavioral health service use among JLIY emerge not from predisposing factors alone, but from their interaction with *enabling factors,* often driven by structural racism, institutional mistrust, and inequitable resource distribution (Dauria et al., 2025; Jordan et al., 2024; Sichel & Elkington, 2023). Enabling factors may include structural and logistical resources (e.g., transportation, financial means, insurance coverage, and availability of services) and family’s capacity, motivation and readiness for services (Barnert et al., 2020; Yonek et al., 2019). Although the JLS may refer youth to behavioral health providers, more pressing needs—along with the burden of scheduling, finding a provider, and navigating costs and insurance—often prevent most JLIY from accessing formal services (Barnert et al., 2020; Dennis et al., 2019; McBrayer et al., 2024). Caregivers and family members are also critical enabling factors, with their involvement consistently ranking among the most reliable predictors of service uptake (Elkington et al., 2020; Gopalan et al., 2010; Yonek et al., 2019). They help set the context for treatment access and often serve as gatekeepers, particularly since parental or caregiver consent is typically required for minors to initiate care (Piper et al., 2024a, b). Additionally, a caregiver’s own history, including their perceptions and experiences with behavioral healthcare, can influence their recognition of a child’s needs and their motivation to seek help (Sheerin et al., 2024, 2025; Yonek et al., 2019).

Finally, *need factors* (e.g., perceived and clinically evaluated need for health services) can be particularly nuanced for this population. Despite the high prevalence of psychiatric disorders among JLIY (Wasserman et al., 2010), youth and caregivers may not view formal treatment as necessary or beneficial (Elkington et al., 2020; McBrayer et al., 2024). These perceptions often reflect broader social and cultural contexts where mental health is less visible or routinely addressed, contributing to lower perceived need for treatment (Misra et al., 2021). In many cases, chronic exposure to trauma, instability, or community violence leads youth to normalize distress, viewing symptoms as a routine part of life rather than a condition that requires treatment (Dye, 2018). Cultural stigma, distrust of systems, and past negative experiences with service providers may also contribute to low engagement, particularly among youth from marginalized backgrounds (Piper et al., 2024a, b; Samuel, 2015; Sichel & Elkington, 2023). Together, these dynamics complicate the pathway to care, even when clinical need is high.

### Alternative and informal pathways to care

To overcome aforementioned barriers to care, JLIY experiencing behavioral health challenges may seek support outside of formal, specialized treatment systems. While most research on youth behavioral health emphasizes conventional clinical interventions (such as outpatient therapy, inpatient hospitalization, and residential programs), far less is known about how JLIY pursue help through non-specialized or informal pathways (McMickens et al., 2024). In many cases, youth receive care in non-healthcare sectors that function as de facto mental health providers. For example, school-based services—delivered by teachers, school counselors, psychologists—or even probation officers in JLS settings are among the most common sources of mental health support outside of clinical environments (Duong et al., 2021). In addition, during acute episodes, some youth may turn to crisis-oriented resources, such as hotlines or emergency departments, for immediate assistance (Aalsma et al., 2017).

Along with these non-specialized sectors, many JLIY rely on informal sources of support, including family members, friends, peers, or members of their faith community (Martinez & Abrams, 2013). Support from community networks may include emotional guidance, practical advice, or structured mentorship from individuals with direct experience of the behavioral health or juvenile-legal systems. For example, programs may connect court-involved youth with trained peer mentors or credible messengers who help families interpret referrals, prepare for appointments, and sustain engagement with services over time (Tolou-Shams et al., 2023). Youth-led peer advocacy and mutual support efforts may also help young people share information about available services, encourage one another to remain engaged in care, and reduce barriers related to stigma or mistrust of formal providers (Creaney, 2020). These forms of support may be perceived by JLIY as more accessible, relatable, and trustworthy than conventional clinical interventions (Klymkiw et al., 2024). However, public health frameworks and surveillance systems often narrowly define what counts as “treatment,” meaning these nonclinical forms of help-seeking are frequently excluded from research and policy discussions (Brown et al., 2014; Rickwood & Thomas, 2012). As a result, patterns of behavioral health service utilization across multiple sectors of care, including formal treatment and informal supports, have not been systematically characterized among justice-involved youth.

#### Present study

To address this gap, this study examined behavioral health service utilization among justice-involved youth across a broader continuum of care than has typically been considered in prior research. Specifically, we examined the service utilization patterns of JLIY across eight distinct sectors of care, ranging from formal behavioral health systems to non-specialist care to community-based supports. We then explored how service seeking patterns were linked to key predisposing, enabling, and need-related factors from the Anderson Behavioral Model of Health Services Use. Our research questions were: (1) what are patterns of JLIY’s behavioral health service utilization across multiple sectors of care, and (2) what are the characteristics (predisposing, enabling, and need factors) of JLIY within each identified pattern of service utilization? By capturing whether and how youth seek care, from specialized treatment programs to schools and family ties, we aim to provide a more comprehensive picture of JLIY’s multiple pathways to care. In doing so, we aim to inform policy and practice by identifying where service systems appear to be functioning as key entry points, and where gaps in coordination exist. We also aim to identify which factors may be most important to target to improve access to timely and appropriate behavioral health care for JLIY.

## Methods

### Study overview

This study used cross-sectional surveys and court administrative data from caregivers of justice-involved youth to examine patterns of youths’ behavioral health service utilization across both formal and informal sectors of care. Specifically, we engaged caregivers of youth participating in a juvenile diversion program who had documented behavioral health concerns. The diversion program operated within a unified family court system that served all adjudicated youth in a Northeastern state. Youth-level data were obtained from court administrative records, and caregivers completed quantitative surveys.

### Procedures

As part of routine diversion protocols, youth were screened for behavioral health concerns by juvenile intake staff using two validated instruments: the Massachusetts Youth Screening Instrument-2 (MAYSI-2; Grisso & Barnum, 2003) to assess mental health and substance use, and the CRAFFT 2.0 (Knight et al., 1999) to assess substance use risk. Prior to their child’s diversion appointment, caregivers were approached by trained research assistants and invited to complete a consent-to-contact form indicating their willingness to be contacted about future research studies for which their child might be eligible.

Caregivers were subsequently contacted by a study team member and were eligible to enroll in the study if their child scored in the clinical “caution” or “warning” range on any MAYSI-2 subscale (e.g., suicidal ideation, depression/anxiety, thought disturbances, somatic complaints, or alcohol/drug use) or screened positive on the CRAFFT. Additional inclusion criteria included being English-speaking and residing with the youth. All study procedures were approved by the [MASKED] Institutional Review Board (IRB).

Data collection occurred between July 2023 and May 2024. At the time of their research appointment, caregivers participated in a virtual session where they provided electronic consent. A trained research assistant facilitated the process, answering any questions and ensuring caregivers understood that participation was voluntary, confidential, and would not affect their child’s legal proceedings. Following consent, the research assistant guided the participants through a survey administered via REDCap software. All appointments were conducted remotely through HIPAA-compliant teleconferencing software (e.g., Zoom). Each session lasted approximately one hour, and caregivers received a $40 gift card as compensation for the survey. At the end of their appointment, all participants were provided with behavioral health resources and referrals as needed. Participants also provided consent for the use of electronic court records to obtain administrative data on youth results of screening instruments and legal system measures.

### Participant characteristics

On average, the 100 youth in our sample were 14.8 years old (SD = 1.54), and 65% were male. Half (50%) identified as belonging to one or more minoritized racial groups, including 23 youth identifying as Black, 5 as American Indian/Alaska Native, 4 as Asian, 2 as Native Hawaiian/Pacific Islander, and 16 as multiracial. In addition, 31.3% identified as Hispanic/Latinx. Household income distribution was as follows: 35.5% earned less than $30,000, 25.8% earned between $31,000 and $60,000, 19.4% earned between $61,000 and $90,000, and 19.4% earned more than $91,000 annually. All youth had health insurance (72% had public insurance and 28% had private insurance). See Table 2 for additional participant characteristics.

### Measures

Caregivers completed a battery of survey measures where they self-reported on their demographics, mental health, substance use, lifetime exposure to traumatic events, and motivation for youth treatment. They also reported on their child’s demographics, mental health symptoms, care utilization, and barriers to treatment participation. Additional information on youth mental health and substance use concerns were extracted from MAYSI and CRAFFT intake assessment scores, which were stored in court records. Youth juvenile legal records (e.g., past system involvement, child welfare involvement) were also drawn from court records.

Key predictors of behavioral health service utilization were based on the Andersen Model of Health Service Utilization (Andersen, 1995), which provides a framework for understanding the factors that influence individuals’ use of health care services. The model categorizes determinants of health service use into three primary domains as outlined in the introduction: predisposing characteristics, enabling factors, and need factors.

#### Behavioral health service utilization

The Child and Adolescent Services Assessment (CASA) was used to capture caregiver-reported youth behavioral health service utilization across eight distinct service settings (Ascher et al., 1996; Farmer et al., 1994). Binary variables were created to indicate lifetime receipt of behavioral health services across the following eight CASA service settings: 1) inpatient treatment (e.g., psychiatric hospital, detox unit), 2) outpatient treatment (e.g., community mental health center, therapist), 3) residential treatment (e.g., group home, rehab), 4) crisis services (e.g., emergency room, crisis hotline), 5) school-based services (e.g., counselor, special education classroom), 6) services from general medical (non-behavioral health specialists) professionals (e.g., primary care provider, social services), 7) services through the JLS system (e.g., probation officer, detention center), 8) and community or informal supports (e.g., family, friends, clergy). Caregivers were asked to indicate whether their child had ever (vs. never) accessed behavioral health services in each service setting. A continuous summary variable was calculated indicating the total number of service settings accessed in the child’s lifetime (range: 0–8).

#### Predisposing factors

Predisposing factors included demographic factors such as youth age, sex at birth, race (coded as 1 = minoritized race or 0 = white), and ethnicity (coded as 1 = Hispanic/Latinè or 0 = not Hispanic/Latinè), prior involvement in the JLS (yes/no), and whether they had current/prior involvement in the child welfare system (yes/no).

#### Enabling factors

Enabling factors included household income (coded as 1 = < $30k, 2 = $31-60k, 3 = $61-90k, and 4 = $91k +) and receiving a court mandate for behavioral health treatment (yes/no). Other enabling factors included caregiver behavioral health, caregiver motivation for youth treatment and caregiver perceived barriers to treatment (scales described below):

##### Caregiver depression

The Patient Health Questionnaire-9 (PHQ-9; Löwe et al., 2004) assessed depressive symptoms (e.g., “feeling down, depressed, or hopeless”). Items were rated on a 0–3 scale, from “not at all” to “nearly every day.” A total score of 10 or higher indicates moderate depression requiring clinical attention. Internal consistency was strong (Cronbach’s alpha = 0.88).

##### Caregiver experiences of trauma

The Life Events Checklist (LEC; Gray et al., 2004) measured lifetime exposure to 16 traumatic events (e.g., accidents, natural disasters, assaults). Participants reported whether they experienced, witnessed, or learned about each event. A total trauma exposure score was calculated by tallying the number of different types of events each participant had been exposed to in their lifetime. Internal consistency was good (Cronbach’s alpha = 0.85).

##### Caregiver alcohol use

Alcohol consumption was assessed using the 10-item Alcohol Use Disorders Identification Test (AUDIT; Saunders et al., 1993), measuring both frequency and negative consequences (e.g., injuring someone due to drinking). A score of 8 + indicated hazardous alcohol use. Internal consistency was strong (Cronbach’s alpha = 0.89).

##### Caregiver motivation for youth treatment

Caregiver Motivation for Youth Treatment Scale (MYTS), a validated 8-item self-report measure that evaluates two key dimensions: problem recognition (awareness and acknowledgment of a behavioral health issue) and treatment readiness (willingness to engage in treatment) (Breda & Riemer, 2012). Each item is rated on a Likert scale ranging from 1 (strongly disagree) to 5 (strongly agree), with higher scores indicating greater motivation. Internal consistency was good (Cronbach’s alpha = 0.86).

##### Barriers to treatment participation scale

The Barriers to Treatment Participation Scale (BTPS) (Kazdin et al., 1997) assessed 38 different barriers to youth treatment participation. Each item is measured on a scale from 1 (strongly disagree) to 5 (strongly agree). The mean score was calculated for the overall BTPS. Internal consistency was excellent (Cronbach’s alpha = 0.92).

#### Youth individual (evaluated) need factors

Need factors encompassed both caregiver- and court staff–evaluated behavioral health needs, as assessed through validated measures described below.

##### Youth pediatric symptom checklist

The Pediatric Symptom Checklist (PSC) was completed by each caregiver. The PSC is a 35-item checklist measuring the number of emotional and behavioral problems in children and adolescents (Jellinek et al., 1988). Caregivers rate each item on a scale of 0 (never), 1 (sometimes), or 2 (often). The total PSC score is calculated by summing all item responses, with a score of 28 or greater indicating clinical significance. The internal consistency was excellent (Cronbach alpha = 0.91).

##### Youth MAYSI-2

The Massachusetts Youth Screening Instrument Version 2 (MAYSI-2) is a brief, standardized mental health screening tool designed for use with justice-involved youth (Grisso & Barnum, 2003). Youth complete the MAYSI-2 during their court intake appointment. The MAYSI-2 consists of 52 yes/no questions assessing symptoms experienced in the past few months. It is structured into seven clinical subscales Alcohol/Drug Use, Angry-Irritable, Depressed-Anxious, Somatic Complaints, Suicidal Ideation, Thought Disturbance, and Traumatic Experiences. Each subscale has a caution cutoff indicating a need for closer attention and a warning cutoff suggesting an immediate need for follow-up. For our study, youth were classified as having a high MAYSI-2 score if they met the warning threshold on one or more subscales.

##### Youth CRAFFT

The CRAFFT 2.0 is a brief, evidence-based screening tool designed to identify substance use and related risks in justice-involved youth (Knight et al., 1999). Youth complete the screening during their court intake appointment. For this paper, we categorized youth as using substances if they reported any use of alcohol, cannabis, tobacco, or other drugs in the past year.

### Data analysis

Descriptive statistics were used to summarize sample characteristics and patterns of behavioral health service utilization. Missing data were minimal (< 5%) and limited to item-level nonresponse on race, ethnicity, and household income variables. Prior to conducting regression analyses, missing values were addressed using multiple imputation via fully conditional specification (FCS).

Latent Class Analysis (LCA) was conducted to identify distinct subgroups of youth based on the eight binary patterns of service use, corresponding to the CASA domains. We investigated LCA solutions with one to four groups. A one group solution would assume that all youth had similar service utilization patterns; and we assumed that solutions with more than 4 groups would have produced groups that were too small for generalization (Nylund et al., 2007). One to four groups models were compared using the Akaike Information Criterion (AIC), Bayesian Information Criterion (BIC), log-likelihood, deviance statistic, and entropy. The three-class model was selected based on optimal model fit, classification certainty (entropy = 0.91), and interpretability (Table 1). The LCA was conducted in R version 4.3.1.

**Table 1:** Comparison of fit statistics, by model

	**log-likelihood**	**AIC**	**BIC**	**Likelihood ratio/deviance statistic (G**^**2**^**)**	**Chi-square goodness of fit (X**^**2**^**)**	**Entropy**
1-Class	−461.1482	938.2965	959.1379	229.2769	775.5738	NA
2-Class	−407.6721	849.3442	893.6321	122.3246	618.2157	0.83
3-Class	−390.6257	833.2514	900.9859	88.23186	260.0367	0.91
4-Class	−385.8021	841.6042	932.7852	78.58463	176.2844	0.79

Following LCA, differences in predisposing characteristics, enabling factors, and need variables across latent classes were assessed using ANOVA for continuous variables and chi-square tests for categorical variables. Multinomial logistic regression models were used to identify factors associated with latent class membership. Predictor variables were selected for model inclusion based on their significance in bivariate comparisons and their theoretical relevance, consistent with the Andersen Behavioral Model. Separate models were estimated for predisposing, enabling, and individual need factors. A final model included all variables simultaneously. Odds ratios (ORs) and 95% confidence intervals (CIs) were reported. Logistic regressions were conducted in SAS 9.4.

## Results

### Behavioral health service utilization

Based on CASA responses, 86% of youth used school-based services, 67% used outpatient services, 61% turned to nonprofessional community members, 59% reported using crisis services at some point in their lifetime, 58% sought help from general healthcare professionals not specializing in behavioral health, 32% accessed JLS services, 26% used inpatient behavioral health services, and 11% accessed residential services. On average, youth used 4 of the 8 (SD = 2.04) different service settings (Table 2).

**Table 2:** Sample characteristics, by class membership

	**Total sample (*n* = 100)**	**Class 1:** **Low intensity, school-centric (*n* = 29)**	**Class 2:** **High intensity, multi-sectoral (*n* = 23)**	**Class 3:** **Moderate intensity, community-based (*n* = 48)**	**Hypothesis tests for differences by class**
	N(%)/m(SD)	N(%)/m(SD)	N(%)/m(SD)	N(%)/m(SD)	F/X2 (*p*-value)
Predisposing characteristics					
Age	14.77 (1.54)	14.59 (1.35)	14.78 (1.41)	14.88 (1.71)	0.62 (0.43)
Sex, Female	35 (35.0%)	9 (31.0%)	9 (39.1%)	17 (35.4%)	0.38 (0.83)
Minoritized Race	50 (50.5%)	16 (55.2%)	12 (54.5%)	22 (45.8%)	0.82 (0.67)
Hispanic/Latinx	31 (31.3%)	14 (48.3%)	6 (27.3%)	11 (22.9%)	5.62 (0.06)
Prior Welfare Involvement	22 (22.0%)	3 (10.3%)	9 (39.1%)	10 (20.8%)	**6.27 (0.04)**
Prior JJ Involvement	22 (22.0%)	4 (13.8%)	7 (30.4%)	11 (22.9%)	2.12 (0.35)
Enabling factors					
Household Income					9.73 (0.14)
< $30k	33 (35.5%)	13 (48.1%)	6 (27.3%)	14 (31.8%)	
$31-60k	24 (25.8%)	6 (22.2%)	8 (36.4%)	10 (22.7%)	
$61-90k	18 (19.4%)	7 (25.9%)	2 (9.1%)	9 (20.5%)	
$91k +	18 (19.4%)	1 (3.7%)	6 (27.3%)	11 (25.0%)	
Treatment Mandate	51 (51.0%)	4 (13.8%)	18 (78.3%)	29 (60.4%)	**24.61 (< 0.001)**
Caregiver Depression	5.54 (5.13)	3.24 (3.70)	7.26 (4.95)	6.10 (5.54)	**4.73 (0.03)**
Caregiver Trauma	4.73 (4.19)	2.83 (4.10)	5.30 (3.95)	5.60 (4.07)	**7.81 (0.006)**
Caregiver Alcohol Use	2.94 (4.48)	2.24 (2.92)	3.73 (6.62)	3.00 (4.06)	0.36 (0.55)
Treatment Motivation	3.63 (0.82)	3.01 (0.79)	4.04 (0.63)	3.80 (0.71)	**15.97 (< 0.001)**
Barriers to Treatment	1.82 (0.59)	2.00 (0.62)	1.74 (0.51)	1.75 (0.59)	1.97 (0.15)
Individual need factors					
Youth PSC Cutoff	54 (54.0%)	7 (24.1%)	20 (87.0%)	27 (56.3%)	**20.57 (< 0.001)**
Youth MAYSI Flag	57 (57.0%)	8 (27.6%)	17 (73.9%)	32 (66.7%)	**14.75 (< 0.001)**
Youth CRAFFT Flag	45 (45.0%)	8 (27.6%)	14 (60.9%)	23 (47.9%)	**6.06 (0.04)**
Service utilization					
Inpatient	26 (26.0%)	3 (10.3%)	23 (100.0%)	0 (0.0%)	**86.02 (< 0.001)**
Outpatient	67 (67.0%)	3 (10.3%)	22 (95.7%)	42 (87.5%)	**59.76 (< 0.001)**
Residential	11 (11.0%)	1 (3.4%)	10 (43.5%)	0 (0.0%)	**32.40 (< 0.001)**
General Medical	58 (58.0%)	2 (6.9%)	20 (87.0%)	36 (75.0%)	**44.70 (< 0.001)**
Crisis	59 (59.0%)	3 (10.3%)	22 (95.7%)	34 (70.8%)	**43.93 (< 0.001)**
School	86 (86.0%)	17 (58.6%)	22 (95.7%)	47 (97.9%)	**25.50 (< 0.001)**
Juvenile Justice	32 (32.0%)	5 (17.2%)	8 (34.8%)	19 (39.6%)	4.25 (0.12)
Community Members	61 (61.0%)	6 (20.7%)	18 (78.3%)	37 (77.1%)	**27.91 (< 0.001)**
Total Service Settings	4.00 (2.04)	1.38 (0.90)	6.30 (1.02)	4.48 (0.87)	**46.67 (< 0.001)**

Bolded values indicate statistical signficance at the *p*<0.05 level

### Latent class analysis

**Fig. 1:**
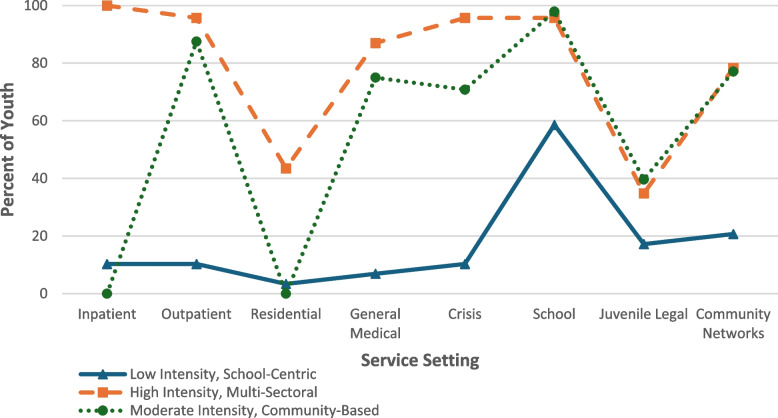
Plot of CASA domains by class membership

We selected the three-class model based on model fit and interpretability. Compared to the one-, two-, and four-class models, the three-class model had the lowest AIC, BIC, deviance statistic, and Chi-square goodness of fit statistic, and the highest entropy (Table 1). The three classes included (1) Low Intensity, School-Centric Service Users (*n* = 29), (2) High Intensity, Multi-Sectoral Service Users (*n* = 23), and (3) Moderate Intensity, Community-Based Service Users (*n* = 48) (Fig. 1).

Class 1, “Low Intensity, School-Centric”, youth were minimal service users, accessing care from an average of 1.38 of the 8 total settings (SD = 0.90). In this class, service utilization across most settings was low (< 20%), with the exception of school-based services, which were accessed by 60% of youth. Class 2, “High Intensity, Multi-Sectoral”, youth engaged with the highest number of service settings, averaging 6.30 (SD = 1.02) settings. They demonstrated consistently high utilization across all sectors, with particularly intensive use of inpatient behavioral health services (100%). Class 3, “Moderate Intensity, Community-Based” youth accessed services from an average of 4.48 settings (SD = 0.87). Their care was primarily delivered through outpatient and other community-based settings, with no utilization of inpatient care.

### Predictors of class membership

Multinomial logistic regression analyses were conducted to understand factors that were associated with latent class membership (Table 3). Separate models were estimated for predisposing factors, enabling factors, and individual need factors. Regression models included variables that were significant in bivariate analyses (Table 2) as well as those identified as theoretically relevant. The full model included key covariates—age, gender, race, ethnicity, and household income—regardless of their statistical significance.

**Table 3:** Multinomial Regression, Predictors of Class Membership

	**Predisposing factors model**		**Enabling factors model**		**Individual need model**		**Full model (predisposing + enabling + need)**	
	Class 2 vs. 1 OR (CI)	Class 3 vs. 1 OR (CI)	Class 2 vs. 1 OR (CI)	Class 3 vs. 1 OR (CI)	Class 2 vs. 1 OR (CI)	Class 3 vs. 1 OR (CI)	Class 2 vs. 1 OR (CI)	Class 3 vs. 1 OR (CI)
Predisposing								
Age	1.05 (0.71, 1.55)	1.07 (0.77, 1.50)	-	-	-	-	1.10 (0.58, 2.01)	1.05 (0.60, 1.82)
Female	1.86 (0.53, 6.5)	1.51 (0.52, 4.36)	-	-	-	-	0.65 (0.09, 4.88)	1.21 (0.25, 5.93)
Minoritized Race	1.09 (0.31, 3.85)	0.91 (0.32, 2.55)	-	-	-	-	1.56 (0.19, 12.53)	0.72 (0.15, 3.44)
Hispanic/Latinx	0.41 (0.11, 1.56)	0.34 (0.11, 1.03)	-	-	-	-	0.33 (0.04, 3.10)	0.22 (0.04, 1.33)
Prior Child Welfare System Involvement	5.30 (1.19, 23.62)*	2.12 (0.52, 8.77)	-	-	-	-	1.86 (0.15, 22.87)	1.08 (0.11, 10.33)
Enabling factors								
Household Income								
< $30k	-	-	ref	ref	-	-	ref	ref
$31-60k	-	-	3.08 (0.44, 21.37)	1.69 (0.31, 9.05)	-	-	3.57 (0.36, 35.70)	1.85 (0.26, 13.25)
$61-90k	-	-	0.15 (0.01, 1.53)	0.34 (0.06, 1.91)	-	-	0.08 (0.004, 1.63)	0.23 (0.02, 2.61)
$91k +	-	-	3.42 (0.09, 123.81)	4.64 (0.16, 132.79)	-	-	3.96 (0.07, 214.37)	4.61 (0.12, 170.81)
MH Mandate	-	-	26.39 (3.87, 180.13)***	10.26 (2.07, 50.95)**	**-**	**-**	34.08 (2.75, 421.68)**	11.30 (1.39, 91.60)*
Caregiver Depression	-	-	1.01 (0.85, 1.22)	1.01 (0.85, 1.19)	-	-	0.94 (0.75, 1.18)	0.94 (0.77, 1.15)
Caregiver Trauma	-	-	1.32 (1.06, 1.65)*	1.31 (1.09, 1.57)**	**-**	**-**	1.38 (1.05, 1.81)*	1.35 (1.07, 1.68)*
Treatment Motivation	-	-	3.64 (1.09, 12.20)*	2.37 (0.93, 6.02)	**-**	-	2.13 (0.41, 11.02)	2.57 (0.69, 9.65)
Need factors								
Youth PSC Cutoff	-	-	**-**	-	23.32 (4.71, 115.33)***	4.48 (1.44, 13.91)**	15.42 (1.31, 181.06)*	2.23 (0.32, 15.29)
Youth MAYSI Flag	-	-	**-**	-	8.92 (2.12, 37.46)**	5.99 (1.99, 18.06)**	10.50 (1.60, 68.90)*	6.39 (1.44, 28.36)*
Youth CRAFFT Flag	-	-	**-**	-	4.63 (1.15, 18.62)*	2.76 (0.89, 8.52)	1.03 (0.14, 7.34)	0.77 (0.15, 3.99)
Model fit stats								
AIC	221.41		184.51		184.91		194.26	
BIC	252.67		226.19		205.75		277.62	
Log-likelihood	197.41		152.51		168.91		130.25	
Pseudo-R2	0.06		0.27		0.20		0.38	

*p *< 0.05*

*p* < 0.01**

*p* < 0.001***

*Bolded values indicate statistical signficance at the p<0.05 level*

In the Predisposing Factors model, youth in Class 2 (“High Intensity”) were significantly more likely to have prior child welfare involvement compared to Class 1 (“Low Intensity”) (OR = 5.30, 95% CI: 1.19–23.62). No other predisposing factors were related to class membership.

In the Enabling Factors model, both Class 2 and Class 3 (“High Intensity” and “Moderate Intensity”) had significantly higher odds of receiving mandated behavioral health treatment (Class 2: OR = 26.39, CI: 3.87–180.13; Class 3: OR = 10.26, CI: 2.07–50.95) and having caregivers with greater trauma exposure (Class 2: OR = 1.32, CI: 1.06–1.65; Class 3: OR = 1.31, CI: 1.09–1.57), compared to Class 1. Additionally, youth in Class 2 had caregivers with the highest levels of treatment motivation (OR = 3.64, CI: 1.09–12.20).

In the Individual Need model, youth in Class 2 and Class 3 (“High Intensity” and “Moderate Intensity”) were more likely to meet the clinical cutoff on the PSC (Class 2: OR = 23.32, CI: 4.71–115.33; Class 3: OR = 4.48, CI: 1.44–13.91), and to have a warning flag on the MAYSI-2 (Class 2: OR = 8.92, CI: 2.12–37.46; Class 3: OR = 5.99, CI: 1.99–18.06). Youth in Class 2 were also more likely to report substance use on the CRAFFT (OR = 4.63, CI: 1.15–18.62).

In the full model, which included all predisposing, enabling, and individual need factors, no predisposing factors remained significant. Predictors of class membership that remained significant in the full model included individual need factors (PSC clinical cutoff, MAYSI warning flag, both highest in Class 2), and enabling factors (mandated behavioral health treatment and caregiver trauma, which were elevated in both Class 2 and Class 3).

## Discussion

**Fig. 2:**
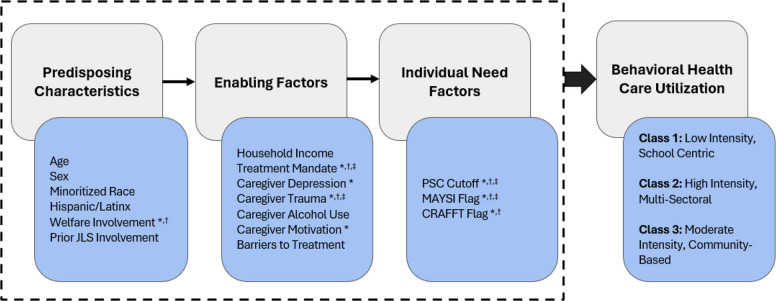
Study findings mapped to andersen behavioral model of health services use. Note: * = indicates factor was significant in bivariate analysis (Table 2), † = indicates factor was significant in partial model (Table 3), ‡ = indicates factor was significant in full model (Table 3)

Understanding patterns of behavioral health service utilization among JLIY is critical to addressing persistent disparities in access to care. Using the Anderson Behavioral Health Model of Service Utilization, we examined predisposing, enabling, and individual need factors associated with different service use patterns. We identified three service use profiles: (1) low intensity school-centric service use, (2) high intensity multi-sector service use, and (3) moderate intensity community-based service use. In this sample, service utilization appeared to be shaped primarily by individual need and enabling factors rather than by demographic predisposing factors (with the exception in the partial model that "High Intensity" service users were more likely to have prior welfare involvement). In the present population, service utilization appeared to be driven primarily by individual need factors and enabling factors. Youth with greater behavioral, psychosocial and substance use needs were more likely to belong to the higher intensity service use groups, and enabling factors, like mandated behavioral health treatment and caregiver motivation for care (Fig. 2), also played important roles in class membership. Taken together, these factors have important implications for how behavioral health services are delivered to JLIY and how juvenile-legal, educational and community systems should be organized and resourced to better support youth before needs escalate.

### Latent class analyses

The identified classes align with prior literature on service use patterns among umbrella populations (e.g. trauma exposed youth, youth with behavioral health concerns, or other systems involving youth) with smaller subsets of JLIY. For instance, in a large nationally representative sample of trauma exposed youth, Choi and colleagues (2018) identified 5 classes of service use patterns (e.g. low service users, child welfare and mental health users, high intensity multisystem users, low intensity multisystem users, and juvenile justice system users) which were closely aligned with those observed here.

Our findings extend this work by specifically focusing on JLIY, an especially vulnerable population whose behavioral health service needs have received limited empirical attention. Although prior research has suggested overlap between JLIY and non-JLIY youth with complex needs (Choi et al., 2018; Duong et al., 2021; Ford et al., 2010), the present study is among the first to delineate within-group differences in service use among JLIY.

### School-based service domain

Our findings show there is a very high reliance on school-based services in our sample (86%), with use of these services being highly prevalent across all three classes. These results align with national trends that show school-based services as the most commonly accessed behavioral health resource for youth (Burns et al., 1995; Duong et al., 2021). In many ways this finding is not surprising. Schools represent one of the most proximal influences on youth outside of their immediate family system and are uniquely situated to provide some of the earliest screening, identification and intervention (Bronfenbrenner, 2005). Such high utilization of the school service domain makes sense given that JLIY are more likely than other groups to exhibit the types of concerns that may be flagged in a school environment (Cauce et al., 2002; Maschi et al., 2008). In fact, schools may be the first place that JLIY’s needs are being identified, as caregiver recognition of youth behavioral health needs often begins with school-related problems, rather than internalizing or externalizing symptoms alone (Pottick et al., 1992).

At the same time, some research has reflected that non-JLIY access school and outpatient service domains at comparable rates (Duong et al., 2021; Farmer et al., 2001). This raises the possibility that JLIY’s disproportionate reliance on school services may reflect limited access to other types of care. Further, JLIY are more likely to hold marginalized identities (Hobaica et al., 2024; Padgaonkar et al., 2021; Simon et al., 2025), and prior research indicates that marginalized youth—who are overrepresented among JLIY—are more likely to access school-based services and less likely to engage with outpatient or inpatient care (Damian & Oo, 2022; Wu et al., 2010).

Our findings underscore how school-based services play a crucial but complicated role for youth in the legal system. While school-based care can reduce racial disparities in service use (Lyon et al., 2013) and serve as a gateway to more intensive treatment (Cauce et al., 2002; Teghethoff et al., 2014), school systems are not equipped to act as primary or sole treatment providers as they are not designed to deliver comprehensive behavioral health treatment (Burns et al., 1995; Cauce et al., 2002; Farmer et al., 2003). These findings highlight the importance of policies that strengthen coordination between schools, juvenile legal systems, and community behavioral health providers, including investments in integrated care systems, school-community referral partnerships, and cross-sector service navigation supports for JLIY. Approaches that encourage structural linkages from schools to specialized behavioral health services may be particularly important for ensuring timely access to appropriate care. Without improved cross-sector coordination, overreliance on school services risks both inadequate care for JLIY and resource strain for schools serving broader populations.

### Multi-sectoral service use

Youth in the high- and moderate-intensity classes engaged with multiple service sectors the most, averaging four and six different settings, respectively. Although multi-sector use may not be the norm, even for all high-need youth (Farmer et al., 2001), it appears more common among JLIY and others facing chronic adversity, including poverty, poly-victimization, and trauma (Choi et al., 2018; Ko et al., 2008; Lyon et al., 2013). Due to the cross-sectional nature of our data, we cannot determine whether multi-sectoral service use as measured here translates to differential treatment efficacy and health outcomes.

Patterns observed in this study may suggest that youth only receive services once their needs escalate to critical levels, requiring simultaneous interventions across sectors. Alternatively, results may reflect limited integration and communication between service systems, such that screening, referral or contact in one sector (e.g., schools, juvenile-legal settings or crisis services) does not consistently result in coordinated follow-up care in another. Importantly, contact with multiple service sectors should not be interpreted as evidence that youth are successfully accessing the most appropriate or effective care. Rather, these patterns may reflect repeated attempts by youth and families to seek support within service environments where referral pathways are fragmented or difficult to navigate. Although these mechanisms cannot be directly evaluated in the present study, several hypothetical but plausible scenarios may help explain these patterns. For instance, youth may be identified as having behavioral health needs in school or court settings but face delays in linkage to outpatient providers. Youth may also cycle between crisis services and community settings without sustained treatment continuity. In some cases, youth may receive treatment mandates through the juvenile-legal system without parallel supports for family engagement or service navigation that facilitate follow-through. Youth may interact with multiple providers across sectors who do not share screening results or treatment plans, limiting coordinated responses to behavioral health needs. It is also possible that there may be differences in eligibility requirements or intake procedures that further disrupt continuity of care even after needs are identified. Alternatively, results may reflect the inability of individual service systems to address co-occurring needs. Prior research has shown that even among JLIY identified as needing behavioral health treatment, only a small fraction initiate or complete care (Belenko et al., 2017; Johnson-Kwochka et al., 2022). As a whole, these results highlight the need for coordinated systems of care for JLIY. Successful interventions for JLIY have emphasized coordination across service domains, family and community systems, and specialized care (Elkington et al., 2020; Maschi et al., 2008). Thus, from a policy perspective, these findings support investments in cross system care coordination, shared referral protocols and integrated service models that reduce fragmentation across juvenile-legal, school, healthcare, crisis, and community-based settings. Such approaches may help ensure that youth with the most complex needs aren’t left to navigate disconnected systems only after their conditions have worsened.

### Anderson model factors

In contrast to much of the existing literature, predisposing factors (e.g., age, race, gender) did not significantly predict service class membership in the full model (Maschi et al., 2008; Spinney et al., 2016; Yonek et al., 2019). While this may reflect sample limitations -including size, recruitment, and the absence of a comparison group—it may also speak to the flattening effects of system involvement itself. Within this sample of JLIY, whose demographic characteristics are shaped by disproportionate surveillance and system intervention, traditional predisposing markers may no longer meaningfully distinguish service access. Instead, service utilization among JLIY in this study was predominately shaped by both individual need factors and enabling factors, both of which are deeply intertwined with structural conditions (Dauria et al., 2025).

 Youth behavioral health needs, such as clinically significant symptoms and flags for behavioral or substance use concerns, were positively associated with both the number and intensity of services used. This finding aligns with prior research demonstrating that clinical need is a strong predictor of service entry (Farmer et al., 2001; Burns et al., 2004). Yet, this relationship is neither automatic nor equitable: 24% of youth in the low-use group (Class 1) screened in the clinically concerning range on the PSC, and 28% had warning flags on the MAYSI and reported substance use on the CRAFFT. While this group had the lowest overall need statistically, a substantial proportion still showed signs of behavioral health concerns yet were not connected to specialized care. These findings suggest a need for policies that strengthen early identification and low-barrier linkage to care so that youth with emerging needs do not remain limited to minimal or school-only supports until symptoms worsen.

Enabling factors, such as caregiver motivation and prior service mandates, played a key role in shaping who received care. These factors may reflect broader systemic dynamics. For example, caregiver motivation for treatment emerged as a key enabling factor, which is notable given caregivers’ central role in facilitating access to services (Djurovic et al., 2024; Rawal et al., 2004; Yonek et al., 2019). This finding highlights the importance of engaging caregivers early in the intervention pathway. Efforts to increase youth service use may benefit from strategies that build caregiver awareness of behavioral health needs, engage caregivers in service decisions, reduce stigma around treatment, and support caregivers in understanding how and where to access appropriate services before needs escalate or court involvement occurs (Piper et al., 2024a, b, 2025). Previous research has also shown that youth symptom severity increases caregiver motivation (Djurovic et al., 2024), a pattern echoed in our findings: youth in the highest-intensity, multi-sectoral service group had caregivers with the strongest treatment motivation. Similarly, youth in higher-use groups (classes 2 and 3) also had caregivers with high levels of behavioral health need. Prior research suggests that caregivers with their own behavioral health challenges are more likely to recognize issues in their children and have the knowledge on how to seek help (Sheerin et al., 2024). From a policy standpoint, these findings point to the need for family-centered models, caregiver navigation supports, and reimbursement of programmatic structures that recognize caregiver engagement as a key component of behavioral health services for JLIY.

By contrast, youth in the lowest-use group (class 1) showed signs of behavioral health needs—at minimum, warranting early intervention—but had the fewest enabling factors to support service use. In particular, they were characterized by lower caregiver motivation compared to the higher-use groups. Another notable difference in this class is the very low reliance on community supports—such as friends, family, and other informal networks—compared to the higher-utilization groups. This pattern is concerning, as community-based social support plays a critical role in promoting mental health and well-being (Stutts et al., 2025). Without these connections, children may miss out on daily support and opportunities for early identification and intervention by non-specialists. This highlights a potential need for family and caregiver-centered interventions aimed at enhancing problem recognition and treatment readiness.

Additionally, youth in higher-use groups were more likely to have received service mandates, which are commonly cited as a primary pathway through which JLIY engage with behavioral health treatment (Janku & Yan, 2009; Maschi et al., 2008; Schwalbe et al., 2009). Youth in the lowest use group (class 1) rarely received treatment mandates, suggesting possible missed opportunities for earlier intervention. However, mandates alone are unlikely to provide a sufficient connection to care (Abrams, 2013; Johnson-Kwochka et al., 2022), and more research is needed to understand how and under what conditions mandates facilitate or hinder access to meaningful treatment. In some cases, even when referrals or mandates are issued, youth and families may encounter low accessibility of services, long waitlists, transportation barriers, or uncertainty about how to navigate available treatment options, which can limit whether referrals translate into sustained engagement with care. These findings suggest that policy responses should not rely on coercive pathways alone but instead should pair referral or mandate processes with broader structural supports (e.g., low-barrier access, community engagement, or culturally-responsive providers). Otherwise, mandates may risk reproducing punitive compliance, rather than meaningful treatment engagement.

Taken together, our findings suggest that behavioral health service use among JLIY is not simply a function of demographic predispositions, but emerges at the intersection of clinical risk and how enabling resources are distributed. Findings also raise concerns about the lack of prevention and early intervention for JLIY: they often do not access specialized treatment services until their needs reach a critical threshold. The high rate of inpatient service use among one subgroup (class 2) may reflect delayed intervention, either due to a lack of support during periods of escalation or an inability of community-based, non-specialized service systems to adequately address youth needs. Due to the cross-sectional nature of the dataset, longitudinal research is needed to examine trajectories into and out of services, the sequencing of service engagement, treatment duration, and the effectiveness of care received.

Broadly, the profiles identified in the study indicate that different groups of JLIY may require different policy and systems responses. Youth in the low-intensity, school-centric group may benefit most from stronger early identification, school-linked referral pathways, and family engagement supports before needs escalate. Youth in the moderate-intensity, community-based group may benefit from policies that stabilize access to outpatient and community services and improve coordination across providers. Youth in the high-intensity, multi-sectoral group may require the most comprehensive systems response, including integrated care planning, cross-system coordination, and timely, sustained access to behavioral health services.

Importantly, community-based supports were among the most commonly used service domains in this sample—particularly among the moderate- and high-intensity service groups—suggesting that families may already be turning to peers, mentors, extended family networks, and community organizations for support and as key pathways to care. These supports may help youth interpret referrals, remain engaged across service transitions, help families navigate intake/eligibility procedures and scheduling barriers or access assistance when formal treatment is delayed or unavailable (Martinez & Abrams, 2013). Support from individuals who have themselves lived through the realities of behavioral health or juvenile legal systems may be particularly important for building trust in services and helping families sustain engagement with care across multiple settings (Creaney, 2020).

Overall, these findings support policies that reduce fragmentation across the juvenile-legal, school, healthcare, and community-based systems and instead promote timely, continuous and developmentally appropriate behavioral health care for JLIY.

### Limitations, strengths, and future directions

This study has several limitations. The relatively small sample size limits our ability to detect small effect sizes, and our purposive sampling of “high-risk” youth with identified behavioral health needs may have reduced variability across several key variables. Additionally, because the sample was drawn from a single juvenile court system and may overrepresent caregivers who were more connected to services or better positioned to participate in research, the findings may not be generalizable to other jurisdictions or to more highly marginalized justice-involved families. The absence of a comparison group restricted our ability to examine disparities based on key factors such as race or socioeconomic status. Finally, the cross-sectional design limits our ability to draw causal inferences. In addition, because service utilization was measured as lifetime exposure across sectors rather than as a sequence of treatment episodes, we were not able to examine how prior negative experiences with providers or service systems influenced subsequent engagement with providers or service systems influenced subsequent engagement with care. However, qualitative findings from a companion study of this sample suggest that mistrust of court-affiliated services, long wait times, limited provider availability and referral navigation challenges may shape treatment engagement across JLIY and their caregivers (Piper et al., 2025).

Despite these limitations, the study's methodology and findings align with those of similar research conducted with larger samples. Future studies should replicate this approach with a larger, more representative sample of JLIY to enhance generalizability and statistical power. Our analysis identified three distinct groups of JLIY based on service utilization patterns and described the types of services most accessible to each. These findings offer a foundation for developing tailored interventions that respond to the specific treatment experiences and needs of different subgroups.

## Conclusion

This study identified three distinct behavioral health service use profiles among JLIY: low-intensity, school-centric users; moderate-intensity, community-based users; and high-intensity, multi-sector users. Utilization patterns were driven primarily by individual need and enabling factors such as symptom severity, caregiver motivation, and court mandates. While school-based services serve as critical access points, overreliance on them may indicate gaps in broader systems of care. Multi-sectoral engagement, though essential for some youth, may reflect unmet needs or fragmented care. To better serve JLIY, interventions and policies should focus on enhancing cross-system coordination, expanding family-centered and community-based supports, and improving access to early, preventive behavioral health care before needs intensify. Longitudinal and comparative research is needed to better understand how service use patterns emerge over time, and how policies and systems may improve treatment access and outcomes for this vulnerable population.

## Data Availability

The data that support the findings of this study are available from the corresponding author, KP, upon reasonable request. Due to the presence of potentially identifiable information, the data are not publicly available to protect participant confidentiality.

## Abbreviations

AIC: Akaike Information Criterion
AUDIT: Alcohol Use Disorders Identification Test
BTPS: Barriers to Treatment Participation Scale
BIC: Bayesian Information Criterion
MYTS: Caregiver Motivation for Youth Treatment Scale
CASA: Child and Adolescent Services Assessment
CI: Confidence Intervals
JLIY: Juvenile Legal Involved Youth
JLS: Juvenile Legal System
LCA: Latent Class Analysis
LEC: Life Events Checklist
MAYSI-2: Massachusetts Youth Screening Instrument-2
OR: Odds ratio
PHQ-9: Patient Health Questionnaire-9
PSC: Pediatric Symptom Checklist
